# The quantitative prevalence of creaky voice (vocal fry) in varieties of English: A systematic review of the literature

**DOI:** 10.1371/journal.pone.0229960

**Published:** 2020-03-11

**Authors:** Katherine Dallaston, Gerard Docherty

**Affiliations:** 1 School of Humanities, Languages and Social Science, Griffith University, Nathan, Queensland, Australia; 2 Arts, Education and Law Group, Griffith University, Nathan, Queensland, Australia; University Hospital Eriangen at Friedrich-Alexander-University Erlangen-Numberg, GERMANY

## Abstract

**Background/aim:**

It is widely believed that ‘creaky voice’ (‘creak’, ‘vocal fry’, ‘glottal fry’) is increasingly prevalent among some English speakers, particularly among young American women. Motivated by the widespread and cross-disciplinary interest in the phenomenon, this paper offers a systematic review of peer-reviewed research (up to January 2019) on the prevalence of creaky voice in varieties of English. The review aimed to understand whose and what speech has been studied, how creaky voice prevalence has been measured, and what the findings collectively reveal.

**Method:**

Literature was located by searching four electronic databases (ProQuest, PubMed, SCOPUS, Web of Science) and the proceedings of two recurrent conferences (‘ICPhS’ and ‘SST’). Studies were included if they reported the prevalence of creaky voice in naturalistic samples of English spoken by vocally-healthy speakers. Reference lists of included studies were cross-checked.

**Results:**

Only ten studies meeting inclusion criteria were identified. All studies sampled a small number of speakers and/or short durations of speech. Nine were recent studies of American-English speakers, and many of these sampled young, female, college students. Across the ten studies, creaky voice was detected using three types of methods, and prevalence was calculated using five different formulae. The findings show that prevalence varies across groups, individuals, and contexts. However, the precise nature of this variability remains unclear due to the scarcity and methodological heterogeneity of the research.

**Conclusions:**

This paper illustrated the application of systematic literature review methods in sociophonetic research—a field in which such methods are not common. The review found that creaky voice prevalence in English is not well understood, and that widespread claims of its recent increase among young American women have not been empirically confirmed. A number of specific limitations in the existing research are highlighted, which may serve as a guide for future research design.

## 1. Introduction

The focus of this paper is the overall rate at which English speakers produce the non-modal voice quality known as ‘creaky voice’, also commonly called ‘creak’, ‘vocal fry’, and ‘glottal fry’. Articulatorily, creaky voice manifests as intervals of low-frequency glottal pulses, which are often characterised by some degree of aperiodicity [1–3]. To the ear, it sounds low-pitched and like “a rapid series of taps, like a stick being run along a railing” [4], or a “rough quality with additional sensation of repeating impulses” [5].

In English, unlike some other languages, phonatory voice quality does not determine the denotational meanings of words or utterances [6]. This means that English speakers are free—from ‘purely linguistic’ constraints, at least—to realise any interval of phonation as creaky voice (or indeed any other voice quality, such as breathy or whispery voice), irrespective of which words are being said. How much phonation is realised as creaky voice may therefore vary, potentially widely, across English speakers. By the same token, the occurrence of creaky voice may vary widely within the same English speaker across different phrasal contexts and communicative situations. The quantifiable amount that speakers produce creaky voice while speaking is here defined as creaky voice *prevalence*.

The prevalence of creaky voice among some English-speaking populations is widely believed to have increased over the last decade, particularly among young American women. Online, numerous opinion articles can be found featuring headlines like *“Vocal fry*: *Women changing voices to sound ‘creaky’ like Kim Kardashian”* [7] and *“Young women*, *give up the vocal fry and reclaim your strong female voice”* [8]. As these headlines suggest, what public commentators tend to debate is not *if* creaky voice is increasingly prevalent among young American women, but *why* and with what social consequence. Mentions of an apparent increase can also be found across various forms of academic publication (e.g. [9, 10, 11 p160]).

Such an increase in creaky voice prevalence—if it has indeed occurred—is of great interest to sociophoneticians, who study speech variability as a function of social, stylistic, and interactional factors at play within spoken communication [12]. The rate at which speakers use creaky voice is also of interest to many speech-language pathologists (SLPs). Despite a general consensus that creaky voice is a physiologically normal phonation type or ‘register’ [6,13], some SLPs hypothesise that its ‘over-use’ can be a symptom of vocal pathology, or lead to its development [14].

Thus, amid the widespread belief that creaky voice is increasingly prevalent among young American women, there is a cross-disciplinary need to disentangle anecdotal claims from empirically demonstrable fact. The purpose of this paper is therefore to clarify what is known—empirically, not anecdotally—about the prevalence of creaky voice in English, how it varies across speakers and situations, and how it may have changed over time. Specifically, through a systematic review of the literature, this paper aims to locate the full breadth of peer-reviewed research on the prevalence of creaky voice in English, describe the methods used to measure creaky voice prevalence, and synthesise the findings. Three research questions (RQs) guide the review:

RQ1: In previous research on the prevalence of creaky voice in varieties of English, whose speech and what speech has been studied?
RQ2: In previous research on the prevalence of creaky voice in varieties of English, how has the prevalence of creaky voice been measured?
RQ3: What do previous studies collectively reveal about the prevalence of creaky voice in varieties of English?

The structure of the paper is as follows. The *Method* section outlines the systematic method used to survey the literature, and provides justification for its use. In the *Results* section, the findings of the review are presented as they pertain to each of the three RQs, in the order stated above. The *Discussion* begins by summarising the state of knowledge, highlighting where knowledge gaps are most pronounced, and why. The paper concludes with a detailed discussion of the methodological limitations of existing research, which may serve as a guide for future research design.

## 2. Method

### 2.1. The systematic approach

The literature on creaky voice prevalence was surveyed using Pickering and Byrne’s [15] systematic approach. The method is explicit and replicable in terms of both the criteria used to determine inclusion, and the strategy used to locate relevant literature. A systematic method was used in place of a more traditional, ‘narrative-style’ method for two reasons. The first is that systematic literature reviews are less vulnerable to invisible biases such as the particular interests and expertise of the author(s). Given the pervasiveness in the authors’ own networks of the above-discussed beliefs surrounding creaky voice prevalence, minimisation of author-related bias, and maximisation of methodological transparency, were prioritised. The second reason for using a systematic method relates to the cross-disciplinary interest in creaky voice prevalence. It was predicted that relevant research may sit in disciplinary ‘silos’, and that an informal or ‘snowballing’ approach may have failed to locate the full breadth of relevant literature.

### 2.2. Criteria for inclusion

The criteria used to assess papers’ suitability for inclusion are detailed below across four sections: (1) terminological criteria; (2) sample criteria; (3) analysis/reporting criteria; and (4) publication criteria. Papers that were not sufficiently detailed to allow for assessment against all criteria were excluded on this basis.

#### 2.2.1. Terminological criteria

Studies were considered relevant if the voice quality under investigation was labelled as any of the following: ‘creaky voice’, ‘creak’, ‘vocal fry’, ‘glottal fry’, ‘pulse(d) register’, ‘pulse(d) phonation’, ‘laryngealized voice’, ‘laryngealization’, ‘glottalized voice’ or ‘glottalization’. The justification for treating each of these terms as essentially synonymous is the pervasive, explicit reference to their synonymy throughout the literature [3,16].

It is acknowledged that a number of ‘creaky-sounding’ phonation (sub)types have been described in the literature, with some taxonomies (e.g. [17]) using some of the terms listed above to distinguish between subtypes. However, delineations of creaky voice subtypes are typically based on the observation that ‘creaky-sounding’ phonation has various physiological underpinnings and/or acoustic manifestations (see [17] for more on this). Whether different creaky voice subtypes are *auditorily* distinct warrants further investigation, but experimental work so far supports the treatment of creaky voice as a unitary auditory phenomenon [18]. Among sociophoneticians and SLPs, creaky voice is overwhelmingly conceptualised as a singular percept.

#### 2.2.2. Sample criteria

Papers were included if their speaker sample and sampling methods met the following inclusion criteria:

1. The speakers must have been ‘native’ speakers of English (i.e. they were not described as ‘English learners’ or ‘L2 English speakers’);
2. The speakers were not purposefully selected for participation based on their use of creaky voice (as they were in [9], for example);
3. The speakers could be assumed to be vocally-healthy (i.e. they were not described as being in poor health or as having vocal pathology);
4. The speech analysed was English (Gibson, Summers and Walls’ [19] study of ‘English-like’ non-words did not satisfy this criterion); and
5. The researchers did not ‘interfere’ with the speakers’ performance by giving instructions regarding their use of creaky voice.

#### 2.2.3. Analysis/reporting criteria

Recall that creaky voice prevalence is defined here as how much phonation is realised as creaky voice. Accordingly, inclusion was limited to studies that analysed whole stretches of continuous speech and coded intervals within as either creaky voice (‘*+Creak*’) or not creaky voice (‘*–Creak*’). Note, however, that this is not the only approach that has been taken to quantifying the ‘creakiness’ of speakers’ voices. One alternative approach is to quantify the ‘creakiness’ of speakers’ voices using ordinal perceptual scale (e.g. as [20] did for speakers of Standard Southern British English); another is to extract acoustic measures like spectral tilt, from which the degree of perceptual ‘creakiness’ (and ‘breathiness’) can be inferred (e.g. as [21] did for speakers of New Zealand English). Such studies are not included in this review because they do not offer an answer to the question *what is the prevalence of creaky voice in English*? Instead, they answer a similar—equally valid, but fundamentally different—question: *how creaky do English speakers’ voices sound*?

The following types of studies were also not included: qualitative accounts of creaky voice use (e.g. [22]); studies that reported the statistical relationships between the occurrence of creaky voice and variables of interest (e.g. sentence length) rather than actual prevalence data (e.g. [23]); and studies reporting prevalence of creaky phonation but only in specific phonemic/prosodic contexts or ‘hand-picked’ words (e.g. [24–26]).

#### 2.2.4. Publication criteria

Finally, inclusion was limited to reports of original research distributed by peer-reviewed academic publications (journals or peer-reviewed conference papers). The purpose of limiting eligibility in this way was to establish a consistent sampling method to identify literature likely to be of a high methodological standard [15]. Reviews, ‘viewpoint’ articles, dissertations, and other research in the ‘grey’ literature were thus not considered eligible. Papers published in languages other than English were not included as their evaluation was beyond the authors’ abilities.

### 2.3. Search strategy and paper selection process

Searches were conducted across four online databases: ProQuest, PubMed, SCOPUS, and Web of Science. These databases were chosen because they index publications from relevant fields—most importantly, from the fields of linguistics and speech-language pathology. No limits were placed regarding year of publication. The searches were first run in April 2018, and repeated in January 2019.

The search strategy for each electronic database is shown in Table 1. Once a preliminary list of keywords was compiled through an informal exploration of the literature, the search strategy was refined by trialling database searches, evaluating the search results, revising the keywords and strategy, and repeating this process. The goal of this iterative process was to decide upon a strategy that would yield as much relevant literature as possible, without returning an impracticable amount of off-topic literature.

**Table 1:** Search strategy to locate literature in four online databases.

Database	Search strategy
ProQuest (search limited to peer-reviewed articles)	ti("voice quality" OR "voice qualities" OR "phonation type" OR "phonation types" OR “vocal quality” OR “vocal qualities”) OR noft("nonmodal phonation" OR "nonmodal voice" OR "non modal phonation" OR "non modal voice" OR "creaky voice" OR "vocal fry" OR "glottal fry" OR "creak" OR "pulsed register" OR "pulsed phonation" OR "pulse register" OR "pulse phonation" OR laryngeali* OR glottali*)
	ti = title noft = anywhere except full text
PubMed	"voice quality"[Title] OR "voice qualities"[Title] OR "phonation type"[Title] OR "phonation types"[Title] OR "vocal quality"[Title] OR "vocal qualities"[Title] OR "nonmodal phonation"[Title/Abstract] OR "creaky voice"[Title/Abstract] OR "vocal fry"[Title/Abstract] OR "glottal fry"[Title/Abstract] OR "creak"[Title/Abstract] OR "pulsed phonation"[Title/Abstract] OR "pulse register"[Title/Abstract] OR (laryngealis[Title/Abstract] OR laryngealization[Title/Abstract] OR laryngealized[Title/Abstract] OR (glottalic[Title/Abstract] OR glottalised[Title/Abstract] OR glottalization[Title/Abstract] OR glottalized[Title/Abstract]
	Title/Abstract = title, collection title, abstract, other abstract and keywords
SCOPUS	TITLE ("voice quality" OR "voice qualities" OR "phonation type" OR "phonation types" OR “vocal quality” OR “vocal qualities”) OR TITLE-ABS-KEY ("nonmodal phonation" OR "nonmodal voice" OR "non modal phonation" OR "non modal voice" OR "creaky voice" OR "vocal fry" OR "glottal fry" OR "creak" OR "pulsed register" OR "pulsed phonation" OR "pulse register" OR "pulse phonation") OR TITLE-ABS-KEY (laryngeali* OR glottali*)
	TITLE-ABS-KEY = Document Title, Abstract, Keywords
Web of Science	TI = ("voice quality" or "voice qualities" or "phonation type" or "phonation types" or "vocal quality" or "vocal qualities") OR TS = ("nonmodal phonation" or "nonmodal voice" or "non modal phonation" or "non modal voice" or "creaky voice" or "vocal fry" or "glottal fry" or "creak" or "pulsed register" or "pulsed phonation" or "pulse register" or "pulse phonation" or laryngeali* or glottali*)
	TI = title TS = topic (title, abstract, author keywords, and Keywords Plus)

Additionally, hand-searches were conducted on the published proceedings of two recurrent conferences: the 4-yearly International Congress of Phonetic Science (‘ICPhS’), from 1999 (the earliest year searchable) to 2015 (the most recent at time of searching); and the biennial Australasian Speech Science and Technology Association’s ‘SST’ conference, from 1986 (the first SST conference) to 2018 (the most recent at time of searching). ICPhS is the flagship international conference for the field of phonetics, with a particularly broad coverage of the field including a significant focus on voice quality. Its online archived proceedings [27] are full papers, with potentially sufficient detail to meet inclusion criteria. The SST proceedings [28]—also full papers—were searched to heighten the likelihood of identifying research on English spoken in the Australasian area, which is relatively less-studied in terms of phonetic variability.

Articles were first screened for relevance by inspection of their title and abstract, if available. A conservative approach was taken during this screening stage—only papers that were very clearly irrelevant (i.e. did not relate to any phonetic aspect of spoken English) were excluded at this point. Then, full texts of the remaining papers were assessed against the criteria for inclusion described above.

Finally, the reference lists of included papers were searched for additional relevant literature. If the title of a referenced paper indicated potential relevance (signaled by use of any of the search terms used in the databases searches; see Table 1) then its full text was assessed for inclusion.

### 2.4. Data extraction

The following data were extracted from the included studies: for RQ1, the description of the speakers and speech material; for RQ2, the method used to detect creaky voice and the ‘formula’ used to calculate its prevalence; and for RQ3, the creaky voice prevalence findings. Data extraction was initially completed by the first author (KD). In order to test the reliability of this extracted data, the second author (GD) then completed a blinded extraction of the same. There was complete agreement with regard to the papers’ creaky voice prevalence findings. However, there were a small number of instances where it was useful for the two investigators to discuss uncertainty arising from the papers’ descriptions of their sampled speakers or of the methods used for detecting creaky voice. These specific instances are noted in the relevant sections below.

### 2.5. Limitations of this method

A limitation of this systematic approach to surveying the literature is that some eligible studies may not have been identified due to them not being indexed by the databases searched, or cited by the included studies. Additionally, the reader is reminded that this review does not include research that might have emerged since January 2019.

## 3. Results

### 3.1. Included studies

**Fig 1:**
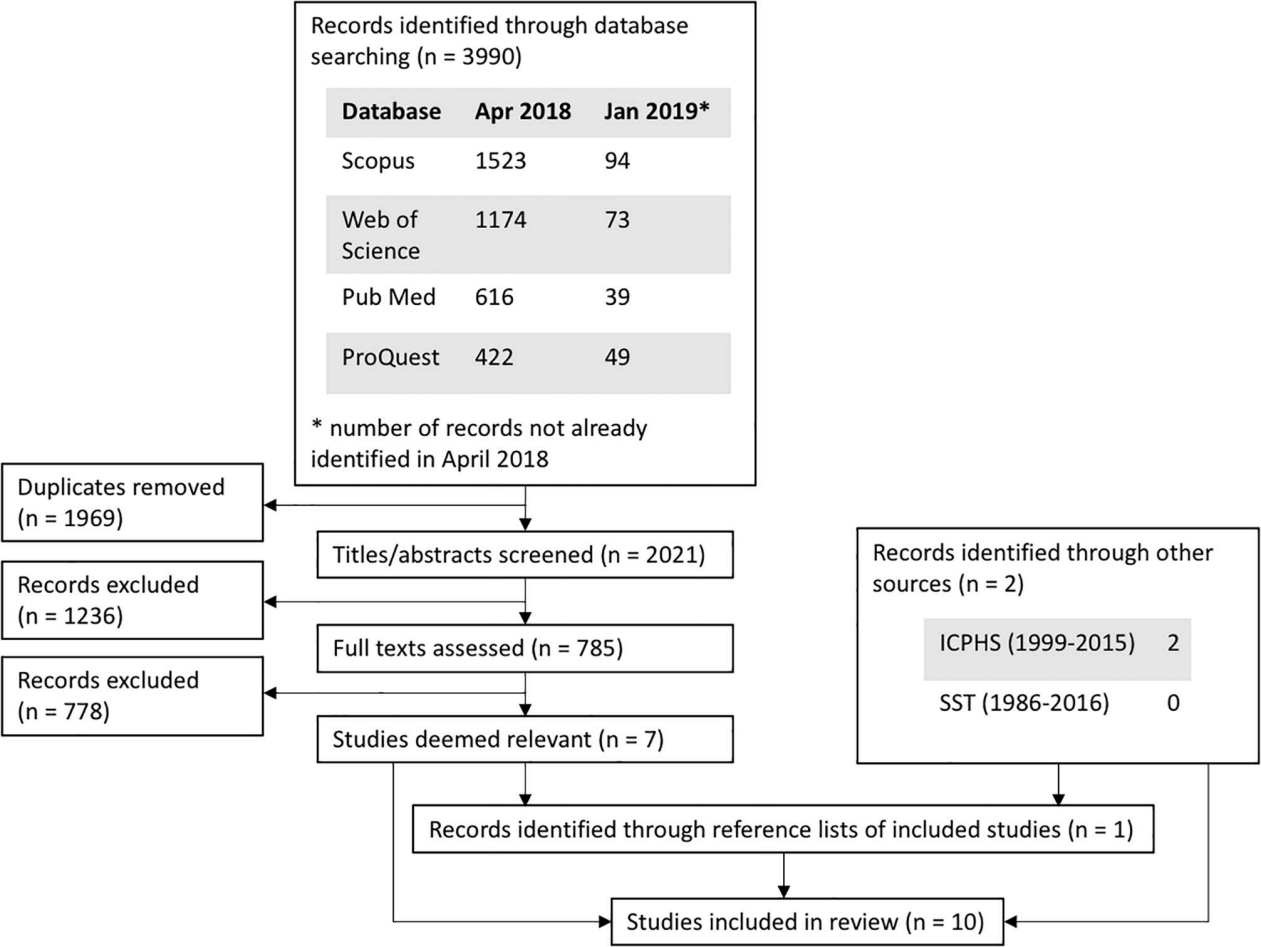
A flow diagram of the search and paper selection process.

A flowchart showing the outcome of the search and paper selection process is provided in Fig 1. Searches of the online databases returned a total of 3990 records. After removing duplicates (n = 1969), the titles and abstracts of 2021 articles were screened. The screening resulted in the exclusion of 1236 articles and the retainment of 785 studies for full-text review (recall that a conservative approach was taken when excluding studies based on their titles/abstracts alone). Inspection of the 785 studies’ full texts found that only seven studies met the criteria for inclusion.

Hand-searches of the ICPhS and SST conference proceedings identified two further studies that met the criteria for inclusion. Note that these two studies were both presented at ICPhS in 2015, share second author, and analysed similarly described speakers and speech material. However, no overlap in their samples was declared, and so none is assumed here. Inspection of the reference lists of the nine included studies (so far) identified one additional study—a book chapter—and inspection of that study’s reference list identified no further relevant studies.

Thus, at the conclusion of the search, this systematic review of literature (published up to and including January 2019) identified only ten studies reporting the prevalence of creaky voice in the speech of vocally-healthy English speakers. These ten studies are listed in Table 2, ordered by year.

**Table 2:** The ten included studies, ordered by year.

First Author	Year	Title
Henton [29]	1988	Creak as a sociophonetic marker.
Yuasa [30]	2010	Creaky voice: A new feminine voice quality for young urban-oriented upwardly mobile American women?
Wolk [31]	2012	Habitual use of vocal fry in young adult female speakers.
Benoist-Lucy [32]	2013	The influence of language and speech task upon creaky voice use among six young American women learning French.
Abdelli-Beruh [33]	2014	Prevalence of vocal fry in young adult male American English speakers.
Luthern [34]	2015	Variation in glottalization at prosodic boundaries in clear and plain lab speech.
Melvin [35]	2015	Gender variation in creaky voice and fundamental frequency.
Oliveira [36]	2016	A comparison of the use of glottal fry in the spontaneous speech of young and middle-aged American women.
Borrie [37]	2017	Conversational entrainment of vocal fry in young adult female American English speakers.
Cantor-Cutiva [38]	2018	Factors associated with vocal fry among college students.

### 3.2. (RQ1) whose speech and what speech has been studied?

#### 3.2.1. Speakers

Table 3 shows a summary of the number of the speakers that participated in each study, and their reported characteristics. Descriptions of the speakers were largely limited to the ‘macrosociological’ [39] characteristics of sex, age, and variety of English. With regards to speaker sex, all of the studies used a binary classification, and none made a distinction between biological sex and self-described gender. Accordingly, the terms ‘females’ and ‘women’, and the terms ‘males’ and ‘men’, are used interchangeably throughout this paper.

**Table 3:** Summary of the samples of the ten included studies.

First Author	Sample size		Age Range	Variety of English
	F	M		
Henton [29]	40^a^	40^a^	25–40^b^	Received Pronunciation & Modified Northern (two accents of British English)
Yuasa [30]	12	11	18–34	American English (California dialects)
Wolk [31]	34	-	18–25	Standard American English
Benoist-Lucy [32]	6	-	20	American English
Abdelli-Beruh [33]	-	34	18–25	Standard American English
Luthern [34]	10	-	18–25	Midland dialect of American English
Melvin [35]	5	5	18–25	Midland dialect of American English
Oliveira [36]	40	-	18–25, 35–50	American English
Borrie [37]	20^c^	-	18–29	American English (Arizona dialect)
Cantor-Cutiva [38]	22	18	20–25	American English (mostly from Midwestern United States)

^a^The second author’s (GD) blinded extraction of data from Henton and Bladon’s [29] study revealed that one speaker was excluded from the analysis due to tape degradation, however the original study did not report the sex or accent of the speaker that was excluded. The findings therefore represent the performance of 79 speakers.

^b^This age range was obtained by consulting the author’s (Henton) 1985 PhD thesis [40] to which readers of the 1988 paper [29] are referred for details of the methodology.

^c^Borrie [37] also reported the prevalence of creaky voice in two speakers who were ‘hand-picked’ to be interlocutors for their experimental study, and who therefore did not meet the inclusion criteria for this review (see Section 2.2.2).

All but one study were analyses of then present-day American English speech (and, as Table 2 shows, all of these studies were conducted this decade). Of all the speakers sampled (n = 297), most were women (64%; n = 189), half were American women (50%; n = 149), and of these American women, at least 55% (n = 82; [31,32,37,38]) were college students aged under 30. Thus, female speech has been studied more than male speech, with a ratio of approximately 3:1, and young adults are more studied than older adults. The most studied English speakers in terms of creaky voice prevalence are young, female, American, college students.

Many of the American English studies described their participants as speakers of a particular variety or dialect of American English (see the last column in Table 3). Of these, Yuasa [30] provided the most detailed description of the sampled speakers’ demographics: all had resided in California for at least five years, but not all were born and raised there, and the group was mixed in terms of speakers’ ethnicities (see Table 1 of [30]). However, in the other American studies, detail regarding the speakers’ residential histories and ethnicities was minimal [32,37,38] or absent [31,33–36].

Henton and Bladon’s [29] study was the only identified study to describe creaky voice prevalence in a non-American variety of English. The study analysed speech recordings from a corpus of British English speakers recorded in the mid-1980s [40] in which speakers were grouped into two accent groups: Received Pronunciation (RP), and Modified Northern (MN). As suggested by Henton and Bladon [29], more information about the speakers and the criteria used to group speakers into the two groups can be retrieved from [40 pp172-175]; in short, the speakers were described as ‘white’ and ‘academically employed’, and grouped based on a perceptual judgement of accent, as well as residential history (the MN speakers had ‘modified’ their accents because after being raised in the area of Leeds, Yorkshire, they had moved to the South of England).

#### 3.2.2. Speech material

Six studies [29,31,33–35,38] analysed read speech only, two studies [30,36] analysed spontaneous speech only, and two studies [32,37] analysed both read and spontaneous speech. Among the eight studies that analysed read speech, the nature and length of the text that participants were asked to read aloud varied. Henton and Bladon’s [29] speakers read aloud eleven stand-alone sentences. Luthern and Clopper’s [34] speakers read aloud five passages, of 35–90 seconds in duration, twice—once in a ‘plain lab speaking style’ and once in a ‘clear lab speaking style’. The names of the passages were not reported in the paper. Melvin and Clopper’s [35] speakers also read aloud five unspecified passages, each approximately 60 seconds in duration, but only once. Benoist-Lucy and Pillot-Loiseau’s [32] speakers read aloud The North Wind and the Sun passage [41], three times. The remaining four studies that analysed read speech [31,33,37,38] had their speakers read The Rainbow Passage [42], but varied in how much of passage they analysed. Borrie and Delfino [37] presumably analysed its entirety, though whether this means just the first six sentences of the passage, as is commonly done in phonetic research, or the whole passage [42], is not specified. The two studies by Abdelli-Beruh and colleagues [31,33] analysed the 2^nd^, 4^th^, and 6^th^ sentences of the passage. Cantor-Cutiva, Bottalico and Hunter [38], whose participants read the passage aloud in nine different simulated noise conditions, analysed only its 6^th^ sentence in each condition.

Among the four studies that analysed spontaneous speech [30,32,36,37], there was also variation in the nature and duration of the speech material analysed. Yuasa [30] recorded her participants having an approximately 10-minute conversation on the topic of food, but analysed only a randomly selected 401-word segment from each conversation. Benoist-Lucy and Pillot-Loiseau [32] analysed 15-minute conversations on a topic of the participants’ choosing; whether the two interlocutors spoke for equal portions of the 15-minutes was not reported. Oliveira et al. [36] analysed monologues of unspecified duration in which participants described all the steps involved in making a peanut butter and jelly sandwich and in doing laundry. Borrie and Delfino [37] analysed speech produced during the first 5 minutes of a cooperative picture-matching task; how much speech was produced by the participants during that 5 minutes (as opposed to that produced by the interlocutor) was not reported.

In the studies that analysed spontaneous, conversational speech (i.e. not a monologue) [30,32,37], there were various controls placed on the conversational partner. In Yuasa’s [30] study, some but not all speakers knew their conversation partner, and all of the women conversed with speakers of the same sex, whereas eight of the eleven men conversed with speakers of the opposite sex. In Benoist-Lucy and Pillot-Loiseau’s [32] study, the (all-female) participants conversed with same-sex interlocutors, all of whom were also participants, and with whom they were familiar. In Borrie and Delfino’s [37] study, the (all-female) participants spoke with two different interlocutors: one woman who had been chosen for her tendency to produce a ‘substantial’ amount of creaky voice while talking, and another woman for her tendency to use a ‘minimal’ amount. Thus, for Borrie and Delfino’s [37] study—a study of ‘conversational entrainment’—the amount of creaky voice in the conversational partner’s speech was a variable of interest.

### 3.3. (RQ2) how has creaky voice prevalence been measured?

Table 4 summarises the methodological characteristics of the studies. Only one study [33] was intentionally designed to use the same the methods as an earlier study [31]; both were conducted by the same group of researchers (Abdelli-Beruh, Wolk and Slavin), and the second study was an ‘extension’ of the first.

**Table 4:** Methodological overview of the ten included studies.

First Author	Detection method			Prevalence Formula				
	1	2	3	1	2	3	4	5
	Auditory	Auditory-visual	Automated Acoustic	% Sentences	% Words	% Syllables	% Speaking time	Number of occurrences per minute
Henton [29]	✓					✓		
Yuasa [30]		✓			✓			
Wolk [31]	✓			✓				
Benoist-Lucy [32]		✓					✓	✓
Abdelli-Beruh [33]	✓			✓				
Luthern [34]			✓^a^			✓		
Melvin [35]		✓				✓		
Oliveira [36]		✓						✓
Borrie [37]	✓						✓	
Cantor-Cutiva [38]	✓			✓				

^a^Luthern and Clopper [34] manually corrected undefined F0 values, but coding decisions were made using automated acoustic criteria.

#### 3.3.1. Methods for detecting creaky voice

Three types of ‘detection methods’ (that is, methods used to decide whether intervals of speech should be coded as *+Creak* or–*Creak*) have been used in creaky voice prevalence studies:

1. *Auditory* methods (used in [29,31,33,37,38]): one or more person listened to the audio recordings and identified the occurrence of creaky voice using pre-determined auditory criteria.
2. *Auditory-visual* methods (used in [30,32,35,36]): like *auditory* methods, but in addition, at some point during analysis, the rater/s visually examined one or more type of acoustic/physiological analysis (e.g. a spectrogram) to assist in coding decisions.
3. *Automated acoustic* methods (used in [34]): an objective, acoustic criterion was used to code speech as *+Creak* or–*Creak*.

In the studies that used *auditory* or *auditory-visual* methods, the auditory criteria for what constituted *+Creak* included: a perceivably low pitch [31,33,35,37]; the perception of a ‘rough’ or ‘gravel-like’ quality [31–33,37]; a train of separately resolvable glottal pulses [29,30,32]; and/or the impression of a ‘popping corn’ sound [30,38]. One study [36] defined creaky voice as “a creaky sound with a rough vocal quality that is produced at a low fundamental frequency” in the paper’s introduction, but did not specify precisely what auditory criteria the raters used during their analysis.

Among the studies that used *auditory-visual* methods, two [32,36] did not explicitly state what constituted visual evidence of creaky voice. However, two did: in those studies, visual evidence of creaky voice was increased irregularity of pulses in the speech waveforms [30,35] and/or widely-spaced vertical striations in a spectrogram representation [30].

The sole study that used an *automated acoustic* method [34] detected creaky voice using the following procedure: after using a forced aligner with manual correction to identify each syllable nucleus, ten equally-spaced fundamental frequency (F0) measures were extracted from each vowel. A syllable was coded as a *+Creak* syllable if the majority of the F0 measures were <150Hz. The justification for this threshold was that it was “based on previous manual coding of voice quality in these data” [34].

#### 3.3.2. Formulae for calculating creaky voice prevalence

The ten included studies calculated creaky voice prevalence using five different prevalence formulae: (note that [32] used two formulae)

1. $\frac{number\;of+Creak\;sentences}{total\;number\;of\;sentences}$ (used in [31,33,38])
2. $\frac{number\;of+Creak\;words}{total\;number\;of\;words}$ (used in [30])
3. $\frac{number\;of+Creak\;syllables\;}{total\;number\;of\;syllables}$ (used in [29,34,35])
4. $\frac{duration\;of+Creak\;}{total\;duration\;of\;speech}$ (used in [32,37])
5. $\frac{number\;of+Creak\;occurences\;}{minutes\;of\;speech}$ (used in [32,36])

In the seven studies that used formulae 1–3 [29–31,33–35,38], the recordings of continuous speech were first partitioned into units: sentences, words, or syllables. Each unit was then coded as either *+Creak* or–*Creak* according to the study’s method for detecting creaky voice (see Section 3.3.1.), and prevalence then calculated as the percentage of units coded as *+Creak*.

In the studies that used formula 4 and/or formula 5 [32,36,37], the whole speech recording (or that portion of the recording selected for analysis) was examined in Praat [43] as a continuous signal, and the boundaries of each interval of creaky voice were annotated in a Textgrid file, according to the study’s method for detecting creaky voice (again, see Section 3.3.1.). For formula 4, the durations (in ms) of all annotated +*Creak* intervals were summed, and prevalence calculated in percentage terms as the total duration of *+Creak* in relation to the ‘total duration of speech’. For formula 5, the number of intervals annotated as *+Creak* were counted, and prevalence calculated as the number of *+Creak* intervals per minute of the speech signal. It was not reported precisely how ‘total duration of speech’ or ‘minutes of speech’ (the respective denominators for formulae 4 and 5) were calculated. Both require a measure of total time spent speaking, but it was not made clear if, for example, momentary pauses between words and phrases were deducted from the calculation.

### 3.4. (RQ3) what do findings reveal?

**Fig 2:**
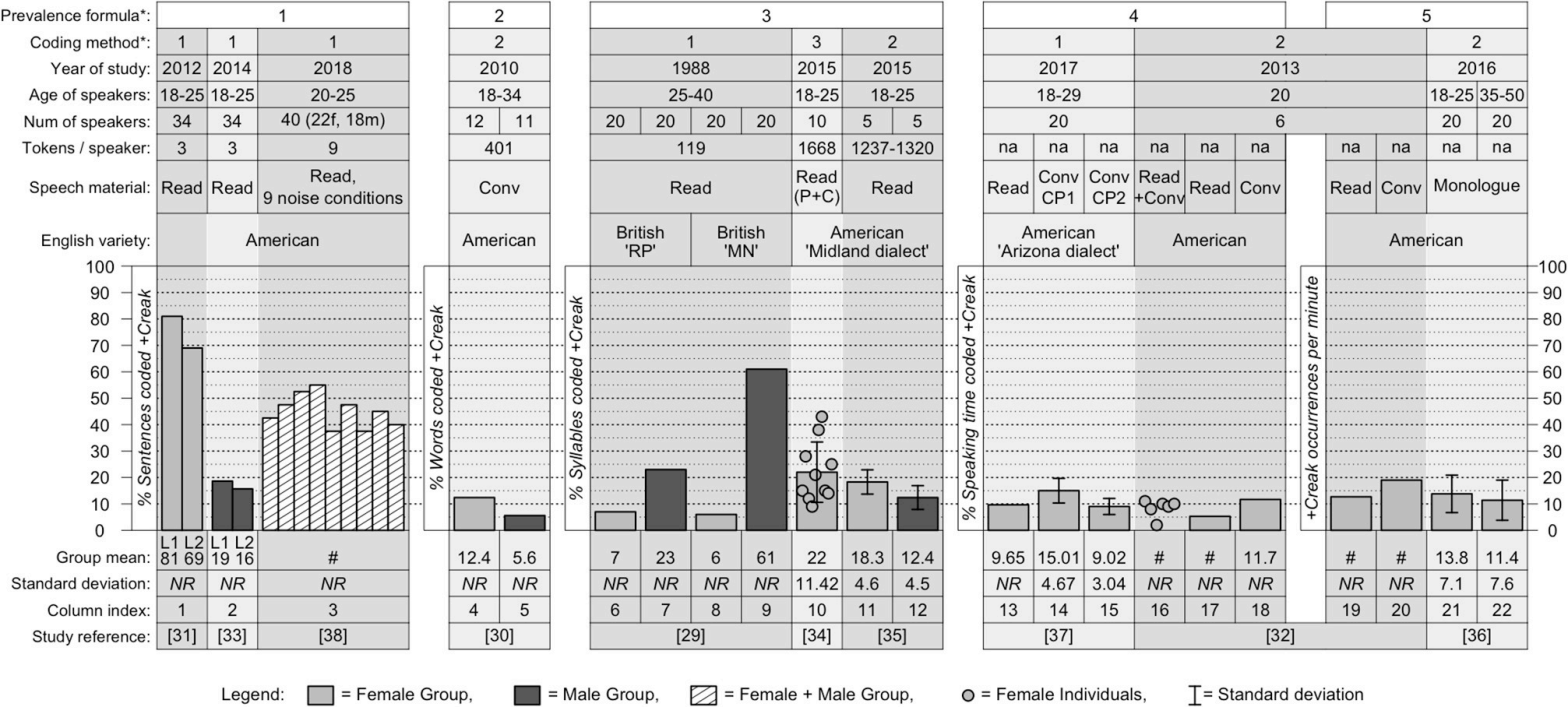
A summary of ten studies’ findings of creaky voice prevalence. * See Table 4 # Exact value not reported. *Abbreviations*: ‘British MN’, modified northern accent; ‘British RP’, received pronunciation accent; ‘Conv’, conversation; ‘CP1’, conversation partner with substantial use of creaky voice; ‘CP2’, conversation partner with minimal use of creaky voice; ‘L1’, listener 1; ‘L2’ listener 2; ‘NR’, not reported; ‘P+C’, combined plain speech and clear speech.

A summary of the ten studies’ findings is shown in Fig 2. Note that the findings of Cantor-Cutiva, Bottalico and Hunter [38] (Fig 2; column 3) and most of Benoist-Lucy and Pillot-Loiseau’s [32] findings (Fig 2; columns 16–17,19–20) are approximate representations of the studies’ results. This is because the data was presented in the original papers in graphs from which only approximate values could be extracted by eye. Hence, a mean and standard deviation has not been calculated for the data in column 16.

Due to the high degree of methodological diversity among the studies (summarised in Table 4), synthesis of the findings is not straightforward. Little insight can be gained by comparing the findings of studies that calculated prevalence using different formulae—this is clear when considering hypothetical questions like: *is ‘50% +Creak syllables’ a higher or lower prevalence rate than ‘50% +Creak words’*? But even between studies that used the same formula, differences in the method used to detect creaky voice confound comparison of results. For example, [29], [34], and [35] all calculated prevalence with the same formula (Fig 2; columns 6–12), but their methods for detecting creaky voice were *auditory*, *automated acoustic*, and *auditory-visual*, respectively. If their detection methods were ‘swapped’, and the data reanalysed, it is not known if the studies would arrive at the same results.

The ten identified studies were thus deemed too methodologically heterogenous to permit a statistical synthesis of their findings (i.e. meta-analysis). In most cases, even a descriptive comparison across studies is inappropriate. Given this, what is presented here is a qualitative synthesis of the studies’ individual results as they pertain to three commonly investigated domains of sociophonetic variation [44], namely: variation across groups; variation across individuals; and intra-speaker variation, across situations/contexts.

#### 3.4.1. Variation across groups

Three studies contribute some insight into how creaky voice prevalence patterns across speakers grouped by age. Oliveira et al. [36] reported the number of creaky voice occurrences per minute in the monologues of 40 American women across two age groups: younger women aged 18–25 (n = 20), and older women aged 35–50 (n = 20). The study found a slightly but not significantly higher rate of *+Creak* occurrences in the speech of the younger women (Fig 2; columns 21–22). Benoist-Lucy and Pillot-Loiseau [32] also reported the number of creaky voice occurrences per minute (also using an *auditory-visual* method) in their sample of 20-year-old American women (n = 6). In comparison to Oliveira et al.’s [36] older group, Benoist-Lucy and Pillot-Loiseau’s [32] 20-year-old speakers produced creaky voice occurrences at a considerably higher rate when conversing with a friend (compare Fig 2; columns 20,22). But see Fig 2, columns 19 and 22—when Benoist-Lucy and Pillot-Loiseau’s [32] speakers were reading aloud, they produced creaky voice occurrences at a similar rate to Oliveira et al.’s [36] older group. Cantor-Cutiva, Bottalico and Hunter [38] conducted multivariate statistical analysis to see whether age was associated with creaky voice use, and found no association. However, this study only sampled speakers aged 20 to 25, arguably a somewhat narrow range for testing age-based sociophonetic differences.

Comparatively more studies have investigated how creaky voice prevalence patterns across speakers when grouped by sex. The studies on American English speakers [30,31,33,35] found that female speakers on average produced more *+Creak* sentences (Fig 2; columns 1–2), more *+Creak* words (Fig 2; columns 4–5), and more *+Creak* syllables (Fig 2; columns 11–12) than male speakers. However, the sole study on British English speakers [29] found an opposite pattern (Fig 2; columns 6–9), and particularly so in the Modified Northern (MN) accent group, in which men produced *+Creak* syllables at a rate approximately 6 times higher than their female counterparts (Fig 2; columns 8–9).

It is tempting to conclude, then, that the prevalence of creaky voice patterns across sex patterns in opposite directions in American English versus British English. However, in comparing the American English studies with the British English study, there are numerous confounding variables. First, the American English studies—those that reveal something about prevalence across sex groups—were conducted in 2010 [30], 2012 [31], 2014 [33] and 2015 [35], and were analyses of then present-day speech recordings; the British English study [29] analysed recordings collected in the mid-1980s. Second, the age ranges of the samples are not the same: the groups of American speakers were aged 18–25 [31,33,35] and 18–34 [30]; the British English speakers were aged 25–40 [29]. A third confounding variable is the range of speech tasks used across the studies (see Section 3.2.2).

#### 3.4.2. Variation across individuals

Five studies reported results in ways that provide insight into the extent of variability across individuals. Two studies [32,34] reported the prevalence scores of individual speakers—in both, the scores reported were pooled scores for two different speaking tasks: Benoist-Lucy and Pillot-Loiseau [32] reported (on a graph, without precise figures) individual scores for read speech and conversational speech combined (Fig 2; column 16); and Luthern and Clopper [34] reported individual scores for two reading styles (‘plain’ and ‘clear’) combined (Fig 2; column 10). Three additional studies [35–37] revealed there was within-group variance by reporting standard deviation (SD) values (Fig 2; columns 11–12, 14–15, and 21–22). Taken together, these five studies show that there is variability across individual speakers in the prevalence of creaky voice, even within relatively homogeneous socio-demographic groupings. However, individual variation was not their focus, and they reveal nothing about *which* individuals deviated far from the group’s mean. Cantor-Cutiva, Bottalico and Hunter’s [38] statistical analysis investigated the association between creaky voice use and a range of individual ‘lifestyle’ and ‘health’ factors, and found some association between speakers’ habitual consumption of caffeine and the occurrence of creaky voice during the final sentence of a reading passage.

#### 3.4.3. Intra-speaker variation, across situations/contexts

Three studies [32,37,38] measured the prevalence of creaky voice across more than one task or speaking context. Benoist-Lucy and Pillot-Loiseau [32] found that, in their sample of 20-year-old American women (n = 6), the average percent of speech produced as creaky voice during conversation was more than double than that while reading aloud (Fig 2; columns 17–18). Borrie and Delfino [37] also found creaky voice to be more prevalent in the conversational speech of young American women (aged 18–29, n = 20) compared to their read speech, but only when their interlocutor was someone who spoke with a ‘substantial’ amount of creaky voice themselves (Fig 2; columns 13–15). (Statistical analysis showed that the extent to which the participants socially aligned with the interlocutor was also a contributing factor—see [37] for more on this). Finally, Cantor-Cutiva, Bottalico and Hunter’s [38] experimental study found that background noise conditions may affect speakers’ likelihood of producing creaky voice (Fig 2; column 3). Together, these three studies suggest that the prevalence of creaky voice tends to be higher in spontaneous speech, but moderating factors include who the interlocutor is, the speakers’ relationship with the interlocutor, and the acoustic conditions in which the conversation is taking place.

## 4. Discussion

The findings of this review can be summarised as follows. Investigations into the prevalence of creaky voice in varieties of English are scarce, modest in scale, extremely methodologically diverse, and lacking in both time depth and geographic breadth. Claims that creaky voice has become increasingly prevalent among young American women are widespread in public and scholarly discourse. However, this systematic review found that such claims are not yet substantiated by quantitative evidence. But nor are they refuted by the evidence. Little is known about the prevalence of creaky voice in varieties of American English prior to 2010, because the first quantitative study of creaky voice prevalence in American English was conducted in 2010, and no studies have analysed historical recordings of American English speakers. Little is known about changes in prevalence *since* 2010, too, because the few studies that have emerged over this period are individually modest in scale (both in number of speakers sampled and amount of speech analysed), and so methodologically diverse that their findings cannot be amalgamated or compared. Furthermore, with regards to a possible increase among young American women in particular, there has been minimal description of present-day older American women’s use of creaky voice, which might offer ‘apparent-time’ evidence of change.

### 4.1. Considerations for future research

The remainder of this paper discusses the implications of this review’s findings for future research on creaky voice prevalence in English. Important considerations are discussed across four aspects of research design: choosing the sample, choosing a detection method, choosing a prevalence formula, and reporting the results.

#### 4.1.1. Choosing the sample

This review found that college-aged American women are the most studied group of English speakers in terms of creaky voice prevalence. This imbalanced focus, which has only amplified over the last decade, seem to be partially driven by beliefs surrounding a recent increase in prevalence in this group; in one of the prevalence studies reviewed herein, the authors explained their decision to sample only American female college students by saying that this “corresponded to the literature consensus stating that the creaky voice effect is more robust among American young women and often occurs in a College context” [32 p2396]. However, this review found that such a consensus is not substantiated with quantitative evidence. In fact, the relationship between beliefs surrounding creaky voice and the design of research seems to be circular; as Lawson [45] has commented, researchers’ focus on creaky voice among young American women has fuelled public discourse on the topic.

Investigations of less studied populations should therefore be prioritised. This includes studies of older speakers, male (or non-binary) speakers, speakers of other English varieties and, if possible, studies of historical speech recordings, too. Because the research also shows that creaky voice prevalence can vary within speakers, across situations and tasks [32,37,38], it is vital that future researchers control for and describe in detail the nature of the speech sample under analysis.

#### 4.1.2. Choosing a detection method

This review found that most prevalence studies have used *auditory* or *auditory-visual* methods to detect occurrences of creaky voice. This is quite justified, given that creaky voice is chiefly a perceptual phenomenon. However, using such methods makes analysis of long speech recordings and/or across a large number of speakers time- and labour- intensive. (In this light, it makes sense that the studies identified by this review were all small in scale). The studies that used *auditory* or *auditory-visual* methods and reported levels of inter- and intra- rater agreement (either formally or informally) reported that agreement was often high, but not complete [29,31,33,36–38]. This is in line with evidence more generally of differences across and within listeners in the perceptual judgement of voice quality [20,46].

The use of *automated acoustic* methods for detecting creaky voice has potential to enable more replicable, larger-scale studies [5,47], but the acoustic complexity of creaky voice [17] means that the reliability of such methods needs close examination. This review found that only one creaky voice prevalence study [34] has made use of such a method. The method used was based on the use of a F0 cut-off point or ‘threshold’ (150Hz) to differentiate between *+Creak* and–*Creak*. Since most manifestations of creaky voice are characterised by low F0 [17], it is conceivable that phonation can be classified as being either *+Creak* or–*Creak*, with a high degree of approximation, using an automated F0-based method [48].

#### 4.1.3. Choosing a prevalence formula

Most studies identified in this review calculated prevalence of creaky voice as the percentage of units of analysis (sentences, words, or syllables) coded as *+Creak*. A ‘unit’-based formula is typical for quantifying the prevalence of allophonic variants of specific phonemes [7]. However, creaky voice is a suprasegmental feature of English [6], so its manifestations do not necessarily align with the boundaries of sentences, words, or syllables. If a researcher does choose to calculate creaky voice prevalence as a percentage of *+Creak* units, they must face the question: *which type of unit (sentences*, *words*, *or syllables–or indeed something else) is best*? One thing to consider is that the ‘size’ (duration) of a unit is inversely proportional to the amount of insight it provides. For example, dissecting continuous speech into large units like whole sentences ‘pixelates’ the data, and gives no insight into how much of each ‘pixel’ is realised as creaky voice; smaller units, such as syllables, give a comparatively higher resolution.

Two other formulae for calculating prevalence were found in the literature. One was the number of *+Creak* occurrences per minutes of speech. This formula arguably offers the least insight in terms of the actual amount that creaky voice occurs during speech; hypothetically, the same amount of creaky voice might manifest as one single occurrence, or several separate occurrences of shorter duration.

The final formula found in the literature was the percentage of speaking time realised as *+Creak*. A strength of this formula is that it would be comparatively less impacted by very short durations of creaky voice associated with glottalised realisations of specific phonemes [24,49]; as for the other formulae, such momentary glottalisations might cause whole syllables, words, or sentences to be coded as *+Creak*. Or they might be counted as whole *+Creak* occurrences. Whether glottalised realisations of specific phonemes ought to ‘count towards’ creaky voice prevalence measures is worth debating. No quantitative creaky voice prevalence study identified by this review explicitly differentiated between very momentary manifestations of creaky voice associated with specific phonemic segments, and suprasegmental manifestations known as ‘phrasal creak’ [50].

#### 4.1.4. Reporting the results

Most studies identified in this review did not report creaky voice prevalence scores for individual speakers. Instead, only means were reported for the whole sample or for sub-groups within the sample. In many cases, not even measures of variance around the group means, such as SD values, were reported. However, studies that did report the findings in a way that revealed within-group variation [32,34–37] showed that creaky voice prevalence varies across individuals, even within relatively narrow demographic groups. Future studies that aim to be optimally informative should therefore report within-group variance whenever possible. Doing so may enable a future meta-analysis.

## 5. Conclusions

As it is, sociophoneticians and SLPs have an incomplete understanding of the prevalence of creaky voice in varieties of English and its variability across and within speakers. The body of research on creaky voice prevalence in English is small and problematically heterogeneous in terms of its methods. Additionally, although the number of studies on the topic has risen in the last decade, so too has an imbalanced focus on young American women. To increase knowledge in this area, this review offers several recommendations, namely: that description of less studied populations should be prioritised; that the potential for automated methods for detecting creaky voice to expand the scope, replicability, and comparability of studies should be explored; that the relative merits of different prevalence formulae used to date should be considered; and, finally, that findings should be presented in such a way that is maximally insightful.

## Supporting information

S1 Data PRISMA 2009 checklist. (PDF)
